# Vancomycin resistance predicts increased mortality in patients with *Enterococcus faecium* bloodstream infections: a six-year experience at a large tertiary care Italian hospital

**DOI:** 10.1093/jac/dkag069

**Published:** 2026-03-06

**Authors:** Lucia Graziani, Tommaso Giani, Alberto Farese, Nicoletta Di Lauria, Elisabetta Mantengoli, Eleonora Riccobono, Gian Maria Rossolini, Alessandro Bartoloni, Michele Spinicci, the ICare Study Group, Marco Allinovi, Giulia Bandini, Lapo Bencini, Stefano Bongiolatti, Manuela Bonizzoli, Luca Bucciardini, Fabio Cianchi, Carlo Di Mario, Ilaria Galeotti, Andrea Galli, Elena Giacomelli, Elisa Giommoni, Stefano Gitto, Gian Luca Grazi, Maddalena Grazzini, Alessandra Ipponi, Regina Maria Lammel, Ilaria Liguori, Lorenzo Livi, Andrea Minervini, Elisabetta Neri, Fabrizio Niccolini, Matteo Piccini, Filippo Pieralli, Paolo Prosperi, Stefano Romagnoli, Mattia Ronchetti, Carlo Rostagno, Bernardo Salani, Massimo Sangiovanni, Sergio Serni, Alessandra Sorano, Pierluigi Stefano, Andrea Ungar, Renato Valenti, Simone Vanni, Silvia Vigiani

**Affiliations:** Infectious and Tropical Diseases Unit, Careggi University Hospital, Florence, Italy; Department of Experimental and Clinical Medicine, University of Florence, Florence, Italy; Microbiology and Virology Unit, Careggi University Hospital, Florence, Italy; Infectious and Tropical Diseases Unit, Careggi University Hospital, Florence, Italy; Infectious and Tropical Diseases Unit, Careggi University Hospital, Florence, Italy; Infectious and Tropical Diseases Unit, Careggi University Hospital, Florence, Italy; Department of Medical Biotechnologies, University of Siena, Siena, Italy; Department of Experimental and Clinical Medicine, University of Florence, Florence, Italy; Microbiology and Virology Unit, Careggi University Hospital, Florence, Italy; Infectious and Tropical Diseases Unit, Careggi University Hospital, Florence, Italy; Department of Experimental and Clinical Medicine, University of Florence, Florence, Italy; Infectious and Tropical Diseases Unit, Careggi University Hospital, Florence, Italy; Department of Experimental and Clinical Medicine, University of Florence, Florence, Italy

## Abstract

**Objectives:**

To report on clinical outcomes associated with vancomycin-resistant *Enterococcus faecium* (VRE) Bloodstream infections (BSIs) observed during a 6-year period at a hospital from an area of high VRE endemicity.

**Material and methods:**

Retrospective study of patients with VRE and/or vancomycin-susceptible *E. faecium* (VSE) BSI in an Italian tertiary care hospital from January 2018 to December 2023.

**Results:**

The cohort included 116 VRE and 225 VSE BSIs. The baseline characteristics were comparable in both populations. Almost half VRE population (53/116, 46%) received no or ineffective empiric therapy against VRE. A targeted effective therapy was initiated with a mean delay of 2.2,  ± 0.4 days in the VRE patients and of 1.2 ± 0.1 days (*P* < 0.01) in the VSE patients. The univariate analysis showed higher rates of septic shock in the VRE group (60% versus 40%, *P* < 0.01), and the 30-day mortality rate was 29% and 46% in VSE and VRE BSIs, respectively (*P* < 0.01). By multivariate analysis, Sequential Organ Failure Assessment score (HR 1.25; 95% CI 1.19–1.31, *P* < 0.001), Charlson Comorbidity Index (HR 1.14; 95% CI 1.06–1.22, *P* = 0.001) and vancomycin resistance (HR 1.93; 95% CI 1.33–2.82, *P* = 0.001) resulted as independent predictors of mortality. The statistical association was confirmed in a sensitive analysis after removing polymicrobial BSI.

**Conclusions:**

*E. faecium* BSIs confirmed to be associated with high mortality rate, especially in fragile patients. Moreover, vancomycin resistance is an independent mortality factor. Further studies are needed to identify patients at higher risk for *E. faecium* BSI.

## Introduction

Enterococci are commensal bacteria of the human intestine frequently involved in severe infections, such as infective endocarditis (IE), urosepsis and bloodstream infections (BSIs). *Enterococcus faecalis* accounts for most of these cases, although the proportion of *Enterococcus faecium* isolates has increased in the last decades.^1–3^ Other species, including *Enterococcus avium*, *Enterococcus gallinarum* and *Enterococcus casseliflavus* have also been isolated from human infections.^4–6^

In recent years, the percentage of multidrug resistant (MDR) strains, namely the vancomycin-resistant enterococci (VRE), surged worldwide, leading the World Health Organization to include VRE among the ‘priority pathogens’ in the global list of antibiotic-resistant bacteria.^7^ According to the surveillance data by the European Centre for Disease Prevention and Control, in Italy VRE percentage among *E. faecium* isolates increased from 11.1% to 32.5% between 2015 and 2023.^8^ On the other hand, the percentage of vancomycin-resistant *E. faecalis* isolates remained stable between 1% and 2% in the same period of time.

VRE can colonize the human gastrointestinal tract, skin and oropharynx. The colonization is associated with some risk factors such as long hospital stay, immunosuppression, frequent hospitalizations, admission to the intensive care unit (ICU) and previous exposure to antimicrobial therapy, including vancomycin and broad-spectrum cephalosporins.^9–10^ Once VRE colonization is acquired, it is persistent in time and leads to a frequent contamination of the environment creating a wide reservoir of VRE that contributes to a rapid spread in hospitals. A recent systematic review and meta-analysis summarized the risk of subsequent infection following MDR bacteria colonization.^11^ In that study, among 4747 VRE carriers, the pooled cumulative incidence of infection at 30 days was 8%, significantly lower than that observed among carbapenem-resistant *Enterobacterales* (CRE) carriers (19%). Nevertheless, BSIs with VRE demonstrated to be burdened by high mortality rates, reported as high as 50%–70%.^12–14^

Treatment options against *E. faecium* are limited, since most strains are resistant to ampicillin and other antimicrobial drugs (intrinsic resistance) and also synergism with aminoglycosides is ineffective (high-level resistance).^1,15^ Vancomycin resistance makes it even more challenging, being linezolid the only approved antimicrobial for the treatment of VRE bloodstream infections to date; on the basis of favourable literature data, other drugs are widely used in clinical practice as monotherapy (e.g. daptomycin)^16–17^ or as combination therapy (daptomycin/β-lactam plus fosfomycin or tigecycline).^18–21^

Despite an increasing interest towards this pathogen, knowledge regarding the epidemiology and clinical impact of VRE infections remains unsatisfactory. Most studies included in reviews and meta-analyses focusing on the role of vancomycin resistance were carried out before linezolid and other effective drugs became available, and under different epidemiological conditions.^14,22^

This paper analyses the characteristics of a 6-year cohort of patients with BSI due to vancomycin-susceptible/-resistant *E. faecium* BSIs at a large Italian tertiary care hospital, aiming to investigate the impact of vancomycin resistance status on clinical outcomes.

## Methods

### Study population

The study retrospectively involved patients with at least one episode of BSI by VRE or by vancomycin-susceptible *E. faecium* (VSE) strain and admitted at the Careggi University Hospital, Florence, Italy, from 1 January 2018 to 31 December 2023.

Eligibility criteria for enrolment were: (i) age ≥18 years and (ii) first episode of microbiologically documented VRE or VSE BSI.

Exclusion criteria were: (i) exitus/discharge in the first 48 hours after BSI onset, (ii) participants who had been already included in the study for a previous VRE/VSE BSI episode and/or (iii) clinical records not available or incomplete.

### Data collection and variables

Demographic, clinical, laboratory, microbiological, treatment and outcome information were collected by reviewing medical records. Data were recorded in a secure electronic sheet.

Pre-enrolment, each patient’s conditions were evaluated through assessment of most important comorbidities and Charlson Comorbidity Index (CCI).^23^ Illness severity at infection onset was assessed through Sepsis-related Organ Failure Assessment (SOFA) score.^24^

Infections were classified as BSIs according to the presence of at least one positive blood culture for VRE or VSE. BSI were classified as polymicrobial when further bacterial and/or fungal pathogens other than VSE/VRE were isolated from the same set of blood cultures or from other sets collected ±72 hours from the index VSE/VRE BSI. We defined a severe acute respiratory syndrome coronavirus 2 (SARS-CoV-2) coinfection when it occurred at the same time as VRE/VSE BSI.

### BSIs and outcome

Data were captured from the electronic medical records and all patients were followed up until hospital discharge or exitus. Survival on day 30 from the day of the first VRE/VSE BSI was assessed for all patients discharged before the end of the follow up.

The primary outcome was 30-day mortality. Secondary outcomes were: ICU admission, time to microbiological cure, recurrency/relapse rate and development of septic shock. Microbiological cure was defined as negative blood culture repeated after ≥72 hours from the first positive sample. In all cases, two to three sets of blood cultures (aerobic and anaerobic bottles) were collected.

Recurrency/relapse was defined as a new documented VRE/VSE BSI after ≥7 days from the first BSI episode and an intervening negative blood culture.

### Microbiological examination

All *E. faecium* isolates were identified by MALDI-ToF (MALDI Biotyper^®^; Bruker Daltonics GmbH, Leipzig—Germany).

The rapid molecular detection of *vanA/B* gene was performed using the BioFire^®^ FilmArray^®^ Blood Culture Identification Panel directly on positive blood cultures.

The susceptibility of the isolates was assessed through commercial broth microdilution (BMD) plates (MERLIN Diagnostika GmbH, Germany). The definition of susceptibility of *E. faecium* strains was based on EUCAST criteria.^25^

In our hospital, rectal swabs are collected every 7 days as part of routine CRE surveillance. The rectal samples were collected with the flocked swab FecalSwab^™^ (Copan, Brescia-Italy) and cultured on selective medium for CRE (CARBA SMART^®^ agar; Biomerieux, Marcy L’Etoile, France), then they were incubated for 24 hours at 37°C. When a grown colony resulted as being compatible with *Enterococcus faecium*, the identification was performed by MALDI-ToF (MALDI Biotyper; Bruker Daltonics GmbH, Leipzig—Germany). The presence of the *vanA* gene, responsible for vancomycin resistance, was confirmed with PCR methodology by means of the Allplex Entero-DR kit (ArrowDiagnostic, Seoul, Korea).

### Statistical analysis

Demographic and clinical characteristics of patients with VSE and VRE BSI were compared by using chi-squared test (or the Fisher exact test, when appropriate) for categorical variables and Wilcoxon test for continuous variables. The Kaplan–Meier method was performed to compare the crude 30-day mortality of patients with VRE and VSE BSI, by using the log-rank test. Data were censored at the end of the 30-day follow-up period from the onset of *E. faecium* BSI or at death. A multivariable Cox regression model including all the variables collected (vancomycin resistance, sex, age, diabetes mellitus, chronic kidney disease, active neoplasm, cardiopathy, CCI, immunodepression, hospitalization in the previous 3 months before BSI, residency in nursing home facilities, concomitant bacteraemia or COVID-19, SOFA score, time between hospitalization and BSI and empiric ineffective antibiotic choice) was performed to identify predictors of 30-day mortality using a backward stepwise procedure (with *P* value <0.2 as significance level for removal from the model). The same analysis was repeated after removing polymicrobial BSI to limit potential bias due to the interference of concomitant infections.

### Ethics

The Local Ethics Committee (registry number 25102) approved the data collection. Informed consent for medical record consultation was obtained from each patient. The study was conducted in agreement with the ethical principles of the Declaration of Helsinki.

## Results

### Demographic and clinical features

**Figure 1.**
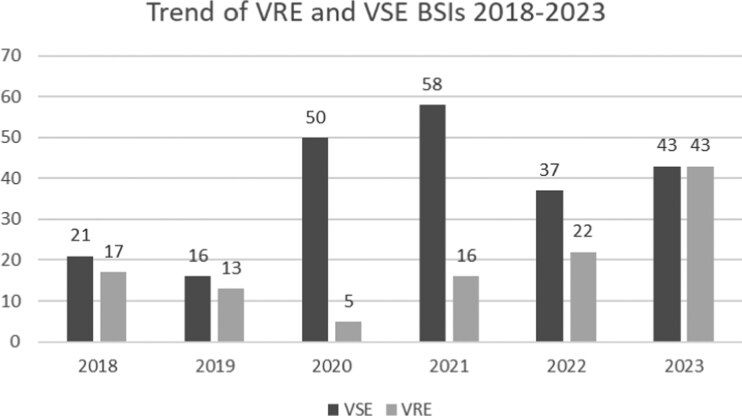
Trend of VRE and VSE BSIs absolute events from 1 January 2018 to 31 December 2023.

We enrolled 341 adult patients with an *E. faecium* BSI, 116 patients (34%) who experienced a VRE BSI and 225 patients who experienced a VSE BSI. The *E. faecium* BSI trend during the observational period is reported in Figure 1.

In both the VRE and VSE populations, more than half patients were male: 63% and 59%, respectively. The median age was 72.5 years (IQR 64–79) in the VRE group and 73 years (IQR 63–80) in the VSE population.

In both groups most patients had a CCI >3 (83% for VRE and 80% for VSE) with a median value of 6 (IQR 4–7), same for both populations. Before hospital admission, the most common underlying condition was cardiovascular disease, observed in 44% of VRE patients and in 39.6% of VSE cases.

The two groups had comparable demographic and clinical characteristics except for the frequency of previous hospital admissions within the 3 months before the index hospitalization, significantly higher in the VRE group than in the VSE group (63.5% versus 45.3%, *P* < 0.01).

Around one-third of patients in both populations had polymicrobial BSI (68/225 and 34/116 in VSE and VRE group, respectively), with a prevalence of Gram-negative infections (73.5% for VSE and 58.8% for VRE). In fact, most polymicrobial infections were diagnosed within ±24 h (95 out of 102 cases of polymicrobial BSI), with only 3 and 4 cases being diagnosed at ±72 h in the VSE and VRE groups, respectively. In the VRE group, 13/34 (38.2%) of the concomitant bacteraemia were sustained by fungi versus 13/68 (19.1%) in the VSE group. In the VSE population the SARS-CoV-2 coinfection was twice more frequent than in the VRE group (17.3% versus 8.6%, *P* < 0.05).

Detailed population characteristics are summarized in Table 1.

**Table 1.** Characteristics of 341 patients with VSE/VRE BSI observed in a 6-year period at a large Italian tertiary care hospital

Variable	VSE (*n* = 225)	VRE (*n* = 116)	*P* value
Epidemiology			
Age, median (IQR), years	73 (63–80)	72.5 (64–79)	0.39
Males (%)	132 (58.7)	73 (63)	0.45
Ward category (%)			
Medical ward	110 (48.9)	63 (54.3)	0.46
Surgical ward	34 (15.1)	18 (15.5)	
ICU	81 (36)	35 (30.2)	
Underlying conditions (%)			
Diabetes mellitus	48 (24)	23 (21.7)	0.64
Cardiovascular disease	89 (39.6)	50 (43.5)	0.49
Chronic kidney disease, CKD	41 (18.2)	22 (19.1)	0.84
SOT/HSCT	10 (4.4)	5 (4.3)	0.98
Haematological malignancy	36 (16)	21 (18.3)	0.60
Solid tumour (active)	53 (23.6)	22 (19.1)	0.35
Immunosuppressive therapy	82 (36.4)	45 (39.1)	0.63
Hospitalization in the previous 3 months	102 (45.3)	73 (63.5)	<0.01
Residence in nursing home facilities	19 (8.4)	15 (13)	0.18
CCI, median (IQR)	6 (4–7)	6 (4–7)	0.69
SOFA score, median (IQR)	4 (2–7)	4 (2–7)	0.51
Timing, median (IQR), days			
Δ date hospital admission, date BSI	15 (7–28)	16 (6–33)	0.51
Δ date VRE rectal colonization, date VRE BSI	NA	6.5 (0–20)	NA
Δ date BSI, effective therapy (mean, ± SD)	1.2, ± 0.1	2.2, ± 0.4	<0.01
In-hospital stay	31 (20–51)	37 (20–56)	0.23
Coinfection and therapy (%)			
COVID-19	39 (17.3)	10 (8.6)	0.03
Polymicrobial BSI^a^	68 (30.2)	34 (29.3)	0.86
Gram+	21 (30.9)	7 (20.6)	
Gram−	50 (73.5)	20 (58.2)	
Fungi	13 (19.1)	13 (54.1)	
Appropriate enterococcal empiric therapy	182 (80.9)	63 (54.3)	<0.01
Rapid blood culture molecular testing	88 (39.1)	42 (36.2)	0.60

^a^Including *S. aureus* (*n* = 5), coagulase-negative Staphylococci (*n* = 19), *Streptococcus* spp. (*n* = 1), *Enterobacterales* (*n* = 56), rods (*n* = 24), *Candida* spp. (*n* = 27), others (*n* = 11).

### Timing of infections and treatment

In the VRE population, 109/116 patients had a documented VRE rectal colonization, while colonization status was unknown in the remaining seven patients. The VRE group experienced the BSI event after a median value of 6.5 days (IQR 0–20) after the first positive rectal swab.

Each patient’s condition at the onset of the BSI was evaluated through SOFA SCORE and in both populations >70% had a SOFA SCORE >2, with a median value of 4 (IQR 2–7).

Concerning empiric antibiotic choice, in the VRE group around half of patients (47.4%) received a drug that was ineffective or they did not receive any antimicrobial therapy, significantly more than VSE patients (18.7%, *P* < 0.001). However, an effective antibiotic therapy, based on the *E. faecium* strain antimicrobial susceptibility, was started within a mean time of 36 h (1.5 days,  ± 0.2) after the BSI event, with a significant delay in the VRE patients (mean 2.2,  ± 0.4 days) compared with VSE (mean 1.2,  ± 0.1 days, *P* < 0.01). The time taken to switch to an appropriate therapy in VRE patients resulted in even longer time periods for those with unknown rectal carrier status when BSI occurred (2.5 days,  ± 0.8) and for those without molecular rapid blood culture testing for vanA/B (2.8 days,  ± 0.7). Even considering coBSI, an inappropriate empiric therapy against the involved isolates other than *E. faecium* did not differ among groups (4/34, 11.7% VRE and 2/68, 3% VSE).

In both groups, linezolid was used in the initial empiric regimen in 30%–35% of cases (VRE 40/116, 34.5%, VSE 69/225, 30.7%). The targeted antibiotic regimens were based on monotherapy in 95% of VRE BSIs (110/116) and in 96% of VSE BSIs (216/225). Linezolid remained the most employed drug (67.2%) for targeted therapy in the VRE group and the second one (40.5%) in the VSE population after vancomycin (44.1%).

Complete data about antibiotic therapy are reported in Table S1 (available as Supplementary data at *JAC* Online) in Supplementary material.

After the BSI event, both VSE and VRE patients had an LOS of >14 days. As shown in Table 1, the overall hospital stay resulted in a median of 37 days (IQR 20–56) in the VRE population and a median of 31 days (IQR 20–51) in the VSE population with no statistical difference (*P* = 0.23).

### Outcome and multivariate analysis

Microbiological cure rates were comparable between the two groups (84.7 and 88.7 in the VSE and VRE groups, respectively), as shown in Table 2. A total of 14 patients had a clinical and microbiological relapse, and 11 of them were in the VSE group.

**Table 2.** Outcome observed in 341 patients with VSE/VRE BSI during a 6-year period at a large Italian tertiary care hospital

Outcome (%)	VSE (*n* = 225)	VRE (*n* = 116)	*P* value
Microbiological cure^a^	127 (84.7)	71 (88.7)	0.39
Days to microbiological cure (median, IQR)	5 (4–9)	6 (3–8)	0.27
ICU admission^b^	19 (8.5)	17 (14.7)	0.08
Septic shock	90 (40)	70 (60.3)	<0.01
Relapse	11 (4.9)	3 (2.6)	0.32
30-day mortality	65 (28.9)	53 (45.7)	<0.01

^a^Microbiological cure available in 150/225 (67%) VSE and 80/116 (69%) VRE patients who had follow-up cultures available.

^b^Following *E. faecium* BSI.

The BSI led to a septic shock in 60% of VRE patients, while in the VSE population this outcome was registered in 40% of the patients (*P* < 0.01) (Table 2).

**Figure 2.**
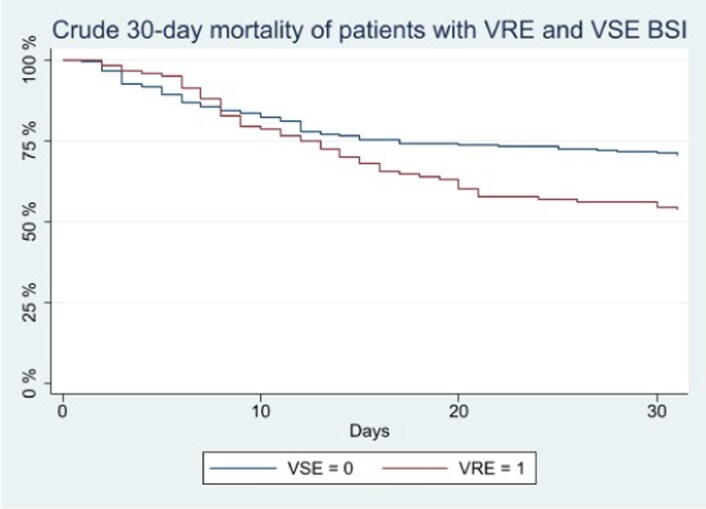
Kaplan–Meier survival estimator of the impact of VRE and VSE BSIs. Log-rank test *P* = 0.006.

The 30-day mortality from the date of BSI event showed a significant difference between the VRE and the VSE groups, with values of 46% (53/116) and 28.9% (65/225), respectively, as shown in the Kaplan–Meier curves in Figure 2 (log-rank test *P* = 0.006).

Four variables were included in the multivariate model by the backward stepwise Cox regression analysis (*P* < 0.2): vancomycin resistance, SOFA score, CCI and SARS-CoV-2 coinfection. Vancomycin resistance (HR 1.93, 95%CI 1.33–2.82, *P* = 0.001), SOFA score (HR 1.25 for each 1-point increase, 95%CI 1.19–1.31, *P* < 0.001) and CCI (HR 1.14 for each 1-point increase, 95%CI 1.06–1.22, *P* = 0.001) were statistically associated with a poor outcome (Table 3A). Vancomycin resistance, SOFA score and CCI were confirmed as predictors of 30-day mortality even after removing 102 patients with polymicrobial BSI from the analysis (Table 3B).

**Table 3.** (A) Predictors of 30-day mortality in 341 patients with VRE/VSE BSI identified by backward stepwise multivariable Cox regression analysis. (B) The same analysis was repeated after removing 102 polymicrobial BSI

(A)			
Variables	Hazard ratio	95% CI	*P* value
Vancomycin resistance	1.93	1.33–2.82	0.001
SARS-CoV-2 coinfection	1.48	0.91–2.43	0.118
CCI^a^	1.14	1.06–1.22	0.001
SOFA score^a^	1.25	1.19–1.31	<0.001

(B)			
Variables	Hazard ratio	95% CI	*P* value
Vancomycin resistance	1.73	1.08–2.70	0.016
CCI^a^	1.12	1.02–1.22	0.010
Male sex	1.38	0.88–2.16	0.160
SOFA score^a^	1.24	1.17–1.31	<0.001

^a^For each 1-point increase.

## Discussion

Today, the burden of VRE infection is increasing worldwide, contributing to the global threat driven by MDR organisms.^2,26^ In Italy, a persistent increase in VRE BSIs was reported from 2015 to 2023, although with significant regional disparities.^27^

In our observational period, VRE BSIs decreased during pandemic years (2020 and 2021) in contrast with European surveillance data,^8^ while we registered an increasing incidence in the following years. This unusual trend might be explained by some factors: increased admission of COVID-19 patients without history of previous hospitalizations, enhanced infection prevention and control measures and consequently reduced nosocomial transmission of VRE,^28^ or a reduced antibiotic selective pressure during the second phase COVID-19 pandemic, for example with a reduction in the empirical use of antibiotics including vancomycin.^29^ These factors could also be responsible for the statistical significance association between SARS-Cov-2 infections and VSE population in our analysis.

Our study showed a 30-day mortality rate as high as 46% in VRE BSIs in line with previous literature data that reported mortality rate from 40% up to 70%.^12–14,30^

*E. faecium* infection itself might be a risk factor for mortality in comparison with other *Enterococcus* spp. infections.^13^ The possible role of secreted virulence factors and cell surface elements as virulence factors of *E. faecium* has been studied. Their accumulation seems to reflect the presence of selective pressure and antibiotic exposure, mostly in nosocomial clades that are usually already carrying antibiotic resistance genes.^31^ According to our findings, vancomycin resistance emerged as an additional factor independently associated with an increased risk of poor outcome, with a 2-fold increase of 30-day mortality risk in the multivariate analysis. Current literature offers conflicting results on the clinical impact of vancomycin resistance among patients with enterococcal BSI.^14,22,32–33^ Three meta-analyses identified an association between vancomycin resistance and an increased mortality in VRE BSIs: in particular, both DiazGranados *et al*.^22^ and Salgado *et al*.^14^ suggested that vancomycin resistance is an independent mortality factor, primarily because patients with a VRE BSI are likely to receive a delayed or ineffective antibiotic therapy against VRE, leading to worst clinical outcome; however, the paper included in the analysis studies conducted before antibiotics currently used as treatment for VRE bacteraemia (e.g. linezolid and daptomycin) have been licensed. A third meta-analysis,^32^ conducted in 2016 after the advent of effective VRE therapy, confirmed the hypothesis of vancomycin resistance as independent mortality factor but it did not definitively estimate whether the mortality excess mirrored the effect of resistance itself or rather different underlying conditions between patients affected by VRE versus VSE bacteraemia. A large cohort study conducted in Germany^13^ in 2018 analysed the possible influence of vancomycin resistance and *Enterococcus* subspecies on in-hospital mortality in enterococci BSIs; in the univariate analysis an association between higher mortality and vancomycin resistance in *E. faecium* was found, together with higher mortality and VSE strains compared with vancomycin-susceptible *E. faecalis* strains; however, the vancomycin resistance role as mortality increasing factor was not confirmed in the multivariate analysis after adjusting for underlying disease, age and *Enterococcus* species. Finally, in the retrospective analysis by Dubler *et al.* the increased mortality in the VRE population was found to be related to acute clinical patients’ conditions and not to vancomycin resistance infections as such.^33^

Other demographic and clinical characteristics associated with increasing mortality included increasing CCI and SOFA score. These findings are consistent with previous studies on VRE BSIs in hospitalized patients.^13,34–36^

Timely introduction of an appropriate antimicrobial therapy is critical for improving the prognosis of patients with severe infections. However, the extent to which timely appropriate empirical and targeted antimicrobial therapies affect mortality in patients with BSI remains unclear. Two recent Italian studies reached opposite conclusions when analysing the impact of delayed therapy on mortality.^30,34^ In a retrospective cohort analysis by Zasowski *et al.* conducted on patients with in-hospital enterococcal BSI, a 48-hour delayed therapy was associated with a 3-fold increase in 30-day mortality; after adjustment for severity of illness and comorbidity the only independent predictor for delayed therapy was vancomycin resistance.^37^ In our cohort, VRE patients were prone to receive an inappropriate empiric therapy in the first instance, and a switch to an effective regimen therapy occurred within a mean of 2.2 days. A rapid correction of empiric therapy was probably fostered by the means of diagnostic and antimicrobial stewardship and infection control activities in place in our hospital. These include fast microbiology tools, a surveillance programme for rectal MDR colonization status (even if with a 7-day window of carrier status unknown) and a dedicated multidisciplinary team supervising the appropriateness of antimicrobial treatments in patients with documented BSI in all services of the hospital. Unsurprisingly, the major delay in administration of appropriate drugs was observed in patients without data about rectal carriage or without molecular testing for vanA/B gene on positive blood cultures. As a result, although initial empiric treatment resulted inappropriate in 46% of VRE BSIs, it was not associated with a 30-day mortality excess, suggesting that a timely correction driven by early microbiological and epidemiological data can make up for an initial ineffective choice.

As for targeted antibiotic regimens, a clear propensity for monotherapy, primarily with linezolid, was observed, both in VSE and VRE BSIs. Daptomycin has been prescribed alone or in combination with other drugs in 46 cases, despite being listed in 2020 EUCAST daptomycin breakpoints for *Enterococcus* species as ‘IE’ for insufficient evidence and advised as needing increased vigilance in the use of high-dose of daptomycin to treat enterococcal BSI.^38^ Considering the high mortality rate reported in the literature as well as in our population, novel trials are warranted to explore different therapeutic approaches, including the role of combination therapies and/or better characterization of the role of drugs other than linezolid in VRE bacteraemia, e.g. daptomycin and long acting lipoglycopeptides.^16,39–40^

Finally, VRE is an emerging leading cause of nosocomial infections and the spread is sustained by human and environment colonization.^9^ However, VRE is probably receiving less attention with respect to other MDR organisms within the infection control programmes worldwide. VRE surveillance activities are heterogeneously implemented, according to a different epidemiology, resources and awareness.^41^ Our data confirmed that surveillance of carriage status by serial rectal swab in inpatients is crucial to adopt infection control measures to prevent nosocomial spread and support the empirical antimicrobial choice in patients with severe infections.

The main limitation of our study is related to the retrospective observational design. A meaningful analysis of the different antibiotic regimen was beyond the study aim, due to the sample size and the characteristics of the study population. Another limitation is that, in our hospital, surveillance for rectal carriage is oriented to CRE, and rates of VRE carriage might have been underestimated since the medium used for screening is not specifically designed for VRE. However, in our experience, most VR *E. faecium* can actually grow on that medium, and all *E. faecium* that grew were microbiologically confirmed as VRE.

Overall, our findings call for a greater attention to the role of VRE infections, as life-threating conditions with high mortality rates, particularly in fragile patients. Vancomycin resistance can be listed as an independent mortality factor. Infection control activities, surveillance for intestinal colonization and fast microbiological support play a crucial role as important allies for a correct management of such patients. Further aids may come from novel clinical score to identify patients at risk to develop invasive VRE infections. Moreover, new trials are warranted to establish more effective therapeutic strategies.

## Supplementary Material

dkag069_Supplementary_Data

## Supplementary data

Table S1 is available as Supplementary data at *JAC* Online.
