# Supplementary material for: Vancomycin resistance predicts increased mortality in patients with Enterococcus faecium bloodstream infections: a six-year experience at a large tertiary care Italian hospital
Source: J Antimicrob Chemother. 2026 Mar 6;81(4):dkag069. doi: 10.1093/jac/dkag069 (PMC13016866; doi:10.1093/jac/dkag069)
Supplement: dkag069_Supplementary_Data [file dkag069_supplementary_data.doc]

**Table S1. Empiric and targeted treatment regimens prescribed in 341 patients with VSE/VRE BSI observed in a 6-year period at a large Italian tertiary care hospital**

| Antibiotic therapy | VSE (n=225) | VRE (n=116) |
| --- | --- | --- |
| Empiric Therapy (%) |  |  |
| Vancomycin  Linezolid  Daptomycin  Tigecycline  Teicoplanin  Daptomycin + Linezolid | 81 (36)  69 (30.7)  20 (8.8)  13 (5.8)  0 (0)  0 (0) | NA  40 (34.5)  10 (8.6)  8 (6.8)  2 (1.8)  1 (0.9) |
| Non effective enterococcal empiric therapy | 42 (18.7) | 55 (47.4) |
| Targeted Therapy (%) |  |  |
| Monotherapy  Vancomycin  Linezolid  Daptomycin  Tigecycline  Ampicillin  Teicoplanin  Linezolid/Vancomycin*  Daptomycin /Linezolid*  Linezolid/Tigecycline*  Vancomycin/Tigecycline* | 78 (34.7)  65 (28.9)  17 (7.6)  16 (7.1)  3 (1.3)  1 (0.4)  17 (7.6)  5 (2.2)  4 (1.8)  4 (1.8) | N/A  69 (59.5)  14 (12.1)  10 (8.6)  0 (0)  0 (0)  0 (0)  4 (3.4)  5 (4.3)  0 (0) |
| Combination therapy  Linezolid + Tigecycline  Linezolid + Daptomycin  Linezolid + Vancomycin  Daptomycin + Tigecycline  Daptomycin + Ampicillin  Vancomycin + Tigecycline | 4 (1.8)  0 (0)  1 (0.4)  3 (1.3)  0 (0.4)  1 (0.4) | 3 (2.6)  2 (1.7)  0 (0)  0 (0)  1 (0.9)  0 (0) |
| Non effective therapy | 6 (2.7) | 8 (6,9) |

* sequential use for clinical decision
 NA: not applicable
